# Fighting Strategies Against the Novel Coronavirus Pandemic: Impact on Global Economy

**DOI:** 10.3389/fpubh.2020.606129

**Published:** 2020-12-09

**Authors:** Bapi Gorain, Hira Choudhury, Nagashekhara Molugulu, Rajani B. Athawale, Prashant Kesharwani

**Affiliations:** ^1^School of Pharmacy, Faculty of Health and Medical Sciences, Taylor's University, Subang Jaya, Malaysia; ^2^Department of Pharmaceutical Technology, School of Pharmacy, International Medical University, Kuala Lumpur, Malaysia; ^3^School of Pharmacy, Monash University, Subang Jaya, Malaysia; ^4^Prin. K. M. Kundnani College of Pharmacy, Mumbai, India; ^5^Department of Pharmaceutics, School of Pharmaceutical Education and Research, Jamia Hamdard, New Delhi, India

**Keywords:** SARS-CoV-2, COVID-19, combination therapy, treatment possibilities, economic impact

## Abstract

Sudden outbreak of a new pathogen in numbers of pneumonic patients in Wuhan province during December 2019 has threatened the world population within a short period of its occurrence. This respiratory tract–isolated pathogen was initially named as novel coronavirus 2019 (nCoV-2019), but later termed as SARS-CoV-2. The rapid spreading of this infectious disease received the label of pandemic by the World Health Organization within 4 months of its occurrence, which still seeks continuous attention of the researchers to prevent the spread and for cure of the infected patients. The propagation of the disease has been recorded in 215 countries, with more than 25.5 million cases and a death toll of more than 0.85 million. Several measures are taken to control the disease transmission, and researchers are actively engaged in finding suitable therapeutics to effectively control the disease to minimize the mortality and morbidity rates. Several existing potential candidates were explored in the prevention and treatment of worsening condition of COVID-19 patients; however, none of the formulation has been approved for the treatment but used under medical supervision. In this article, a focus has been made to highlight on current epidemiology on the COVID-19 infection, clinical features, diagnosis, and transmission, with special emphasis on treatment measures of the disease at different stages of clinical research and the global economic influence due to this pandemic situation. Progress in the development on vaccine against COVID-19 has also been explored as important measures to immunize people. Moreover, this article is expected to provide information to the researchers, who are constantly combating in the management against this outbreak.

## Introduction: Coronavirus

**Figure 1:**
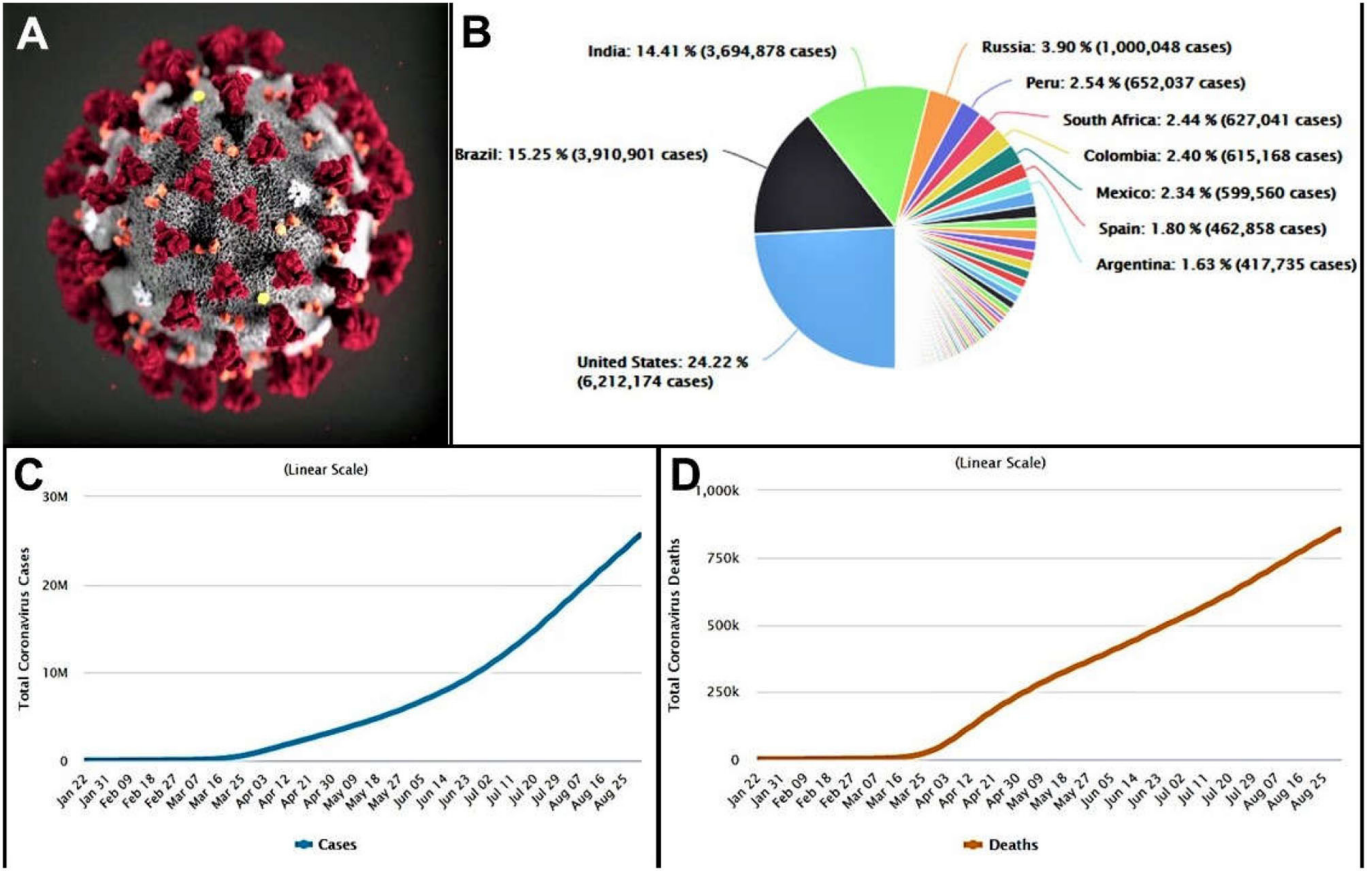
**(A)** Morphology of the coronavirus (CDC). **(B)** Country-wise distribution of COVID-19 cases. **(C)** Total active cases of coronavirus infected people. **(D)** Total deaths of coronavirus infected patient. (data obtained on, September 1, 2020 from https://www.worldometers.info/coronavirus/).

The term “corona” has become a global threat nowadays, which has changed the world and our lives completely. The microscopic pathogen creates a circumstance, against which scientists are continuously trying to find an effective remedy. This coronavirus (CoV) was first identified in 1965 by Tyrrell and Bynoe; however, initially, this virus was named B814 for identification (1). Within the same time frame, Hamre and Procknow reported the growth of viruses with unfamiliar properties from the respiratory samples of cold-infected students (2). Simultaneous research by Almeida and Tyrrell revealed that the viruses identified by the previous groups were of similar morphology as evidenced by the electron microscopic representations. It was also revealed that these pleomorphic membrane-coated particles are of 80–150 nm in size containing the covering of widely spaced club-shaped surface projections (3). Later, in the late 1960s, this new group of the virus was named as “coronavirus” by Tyrrell and colleagues, because of the presence of crown-like surface appendages or spiked projections under the electron microscope, when they were investigating on different strains of human and animal viruses (Figure 1A) (4, 5). Further investigations discovered that these enveloped non-segmented viruses consist of a single-strand, positive-sense RNA genome (size 26–32 kilobases), which belong to the Coronaviridae family (order: Nidovirales) (6, 7). These CoVs are broadly distributed in humans, mammals, and birds to cause several health problems, largely respiratory, neurologic, hepatic, and enteric diseases (8, 9). Earlier to 2019, there were six species of CoV known to cause global challenges for public health, where the prevalent four species are human-CoV (HCoV) 229E, HCoV-OC43, HCoV-NL63, and HCoV-HKU1, which are responsible for causing mild human symptoms related to the common cold in immunocompetent individuals (7, 10). The other two beta-CoV species, severe acute respiratory syndrome CoV (SARS-CoV), and Middle East respiratory syndrome CoV (MERS-CoV) are of zoonotic in origin and resulted in epidemics with more than 10,000 cumulative cases (11). Among these two, the fatal rate of MERS-CoV (in 2012 in the Middle East) was higher (37%) compared to SARS-CoV (in 2002 and 2003 in China) (10%) (12, 13), where both the species were the causative agent for the severe respiratory disease (8).

In December 2019, a novel CoV (initially termed 2019-nCoV) infecting pneumonia broke out in more than 100 countries over the five continents, which was first reported in Wuhan, Hubei Province, China (8, 14). Later, this 2019-nCoV has been renamed SARS-CoV-2 by the International Committee of Taxonomy of Viruses (15). At the time of writing, on September 1, 2020, a total of 25,662,091 cases of SARS-CoV-2 infections in 215 countries were reported worldwide with a sharp trend to raise the numbers (Figure 1B). Of the infected cases reported, 18,817,777 cases were closed, with 17,962,425 cases of recovery (95%) and 855,352 cases of deaths (5%) (Figures 1C,D) (16). SARS-CoV-2 is also enveloped, single-stranded RNA beta-CoV, similar to MERS-CoV and SARS-CoV, where the genome encodes structural spike glycoprotein and non-structural papain-like protease, 3-chymotrypsin–like protease, helicase, and RNA-dependent RNA polymerase and auxiliary proteins (17).

Considering the existence of various species of animal CoVs, it is not surprising to accept a novel species to cause a severe problem in the respiratory system, SARS-CoV-2, emerged in a part of China and extends its severity throughout the world. This virus was grown in culture medium easily to enable the genome sequencing in order to correlate with existing CoV (18). In a recent report of SARS-CoV-2, it has been revealed that the sequencing of SARS-CoV-2 differed sufficiently from the known human CoVs reported earlier; there is 79.6% identity to the whole genome sequence between SARS-CoV-2 and SARS-CoV (19). Alternatively, Zhu and colleagues revealed that SARS-CoV-2 is a distinct particle from the SARS-CoV and MERS-CoV comparing the full-length sequencing and phylogenic analysis (8). Finally, relating the genome sequence analysis for the nonstructural protein domains, it has been recommended that this novel species of CoV belongs to SARS-CoV (19). The genome sequence of SARS-CoV-2 is found to be quite similar to the animal CoVs, where there is a 96% similarity to the bat CoV (19). In continuation, Zhu et al. mentioned that SARS-CoV-2 has a close similarity to bat CoV (8) as reported by the earlier group. Based on this fact, it has been postulated that the primary source of this infection is bats; however, investigations are still ongoing to report the actual source of transmission of SARS-CoV-2 to result in a pandemic.

## Clinical Characteristics of COVID-19 Patients

CoV disease 2019 (COVID-19) has brought a cluster of pneumonia cases in hospitals worldwide. Different researchers have presented the clinical features of the COVID-19–infected patients, which is summarized in this section of the article. Chen and colleagues have reported pneumonia infected 99 cases of COVID-19 patients, where the average age of the patients was 55.5 ±13.1 years. Of the 99 patients, acute respiratory distress syndrome (ARDS) was reported in 17 patients (17%) within a short period, where 11 (11%) of them died because of multiple organ failure. At the time of reporting, 57 (58%) of the infected patients were hospitalized for recovery of their symptoms. The clinical manifestations of these patients are presented in Table 1 (20). Similarly, Huang and colleagues reported the clinical features of this disease in 41 hospitalized patients with COVID-19, with a median age of 49 years. The clinical manifestations are quite similar to the previous report by Chen and colleagues. A total of 29% (12 patients) acquired ARDS, whereas 12% (five patients) acquired cardiac injury, 7% (three patients) acquired shock, and 7% (three patients) had acute kidney injury. Compared to the previous reports, the percentage of deaths in COVID-19 patients was more in this report [6 (15%)] (11).

**Table 1:** Representation of clinical manifestations of COVID-19 patients.

**Fever**	**Cough**	**Phlegm**	**Anorexia**	**Shortness of breath**	**Sputum production**	**Muscle ache/fatigue**	**Confusion**	**Headache/ dizziness**	**Sore throat**	**Chills**	**Hemoptysis**	**Rhinorrhea/ nasal congestion**	**Chest pain**	**Diarrhea**	**Abdominal pain**	**Nausea and vomiting**	**References**
83%	82%			32%	—	11%	9%	8%	5		—	4	2%	2%		1%	(20)
98%	76%			55%	28%	18%	—	8%	—		5%	—	—	3%		—	(11)
98.6%	59.4%		39.9%	31.2%	—	34.8%/69.6%	—	9%/9.4%	—		—	—	—	10.1%	2.2%	10.1%	(21)
96%	47%	20%	18%	14%		31%		16%				6%		10%		6%	(22)
88.7%	67.8%			18.7%	33.7%	14.9%/38.1%		13.6%	13.9%	11.5%	0.9%	4.8%		3.8%		5%	(23)

Further reports on 138 COVID-19 diseased patients provided more insights on the clinical symptoms of the pneumonia-infected patients. The median age of the patients was 56 years; the clinical manifestations of the patients are displayed in Table 1. The report also included a decrease in lymphocyte counts in 70.3%, prolongation of prothrombin time in 58%, and increased lactate dehydrogenase in 39.9% of patients (21). Of the 138 COVID-19 patients, 36 patients (26.1%) were transferred to the intensive care unit because of ARDS [22 (61.1%)], arrhythmia [16 (44.4%)], and shock [11 (30.6%)]. While reported, 47 patients were discharged alive (34.1%), with a mortality rate of 4.3% (6). However, the rest of the patients were still hospitalized for their recovery (21). Continuing clinical features of 51 COVID-19 patients with an average age of 49 ± 16 years are depicted in Table 1. The symptoms of the patients are quite similar to earlier reports (22). Guan and colleagues presented a larger extracted data from 1,099 patients affected with COVID-19 recently with a median age of 47 years. The reports described the median incubation period for this virus as 4 days (interquartile range, 2–7) with a mean hospitalization period of 12.8 days. The mortality rate of this study (1.4%) was much less than the previous reports, which might be due to the number of cases and inclusion criteria of the present study (23). From the available reports in COVID-19, it is clear that SARS-CoV-2 is a combination of clinical manifestations where older patients and the patients with comorbidities are prone to reach to the fatal respiratory condition due to ARDS.

**Figure 2:**
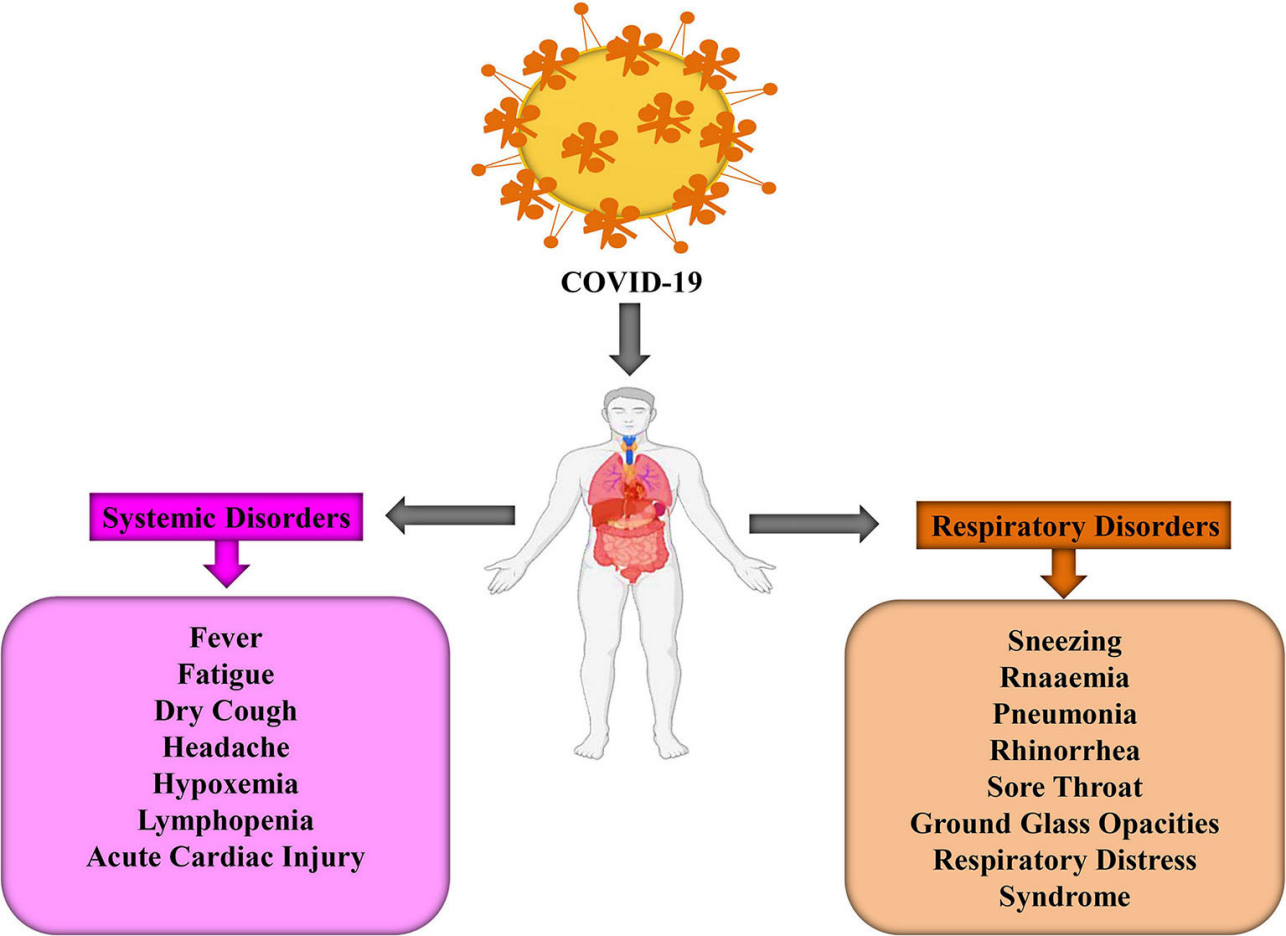
The systemic and respiratory disorders caused by COVID-19 infection (24).

Different systemic and respiratory disorders related to COVID-19 have been picturized in Figure 2. Following the incubation period, these virus-infected persons started showing symptoms similar to beta-CoV; however, SARS-CoV-2 is grossly creating life-threatening conditions when invading the lower respiratory tract (25). Therefore, protecting individuals from exposing themselves in the contaminated environment, taking preventive measures from the transmission modes, and, finally, proper diagnosis of the suspected patients and immediate treatment strategies could protect patients from worsening toward fatal condition.

## Diagnostic Pathways for the Invading SARS-COV-2

Diagnosis of SARS-CoV-2 infection is critical to respond effectively for the proper treatment of the patients. The increasing number of patients worldwide warrants a proper guideline for diagnosing the patients at their early stages for the treatment.

### Physical and Clinical Examinations

Apart from the clinical symptoms, a physical examination may help in the diagnosis of the disease at an advanced stage because the mild signs are not presenting positive signs at the early stage of the disease. At an advanced stage, the SARS-CoV-2–infected patients may show shortness of breath, weakened breath sounds, moist rales in lungs, decreased or increased tactile speech tremor, and dullness in percussion (26).

The clinical manifestations of the SARS-CoV-2–infected patients have already been discussed in the earlier section, where fever, dry cough, and fatigue are the typical symptoms to diagnose; however, biases may interfere into the proper diagnosis, which might require subsequent radiological imaging and laboratory tests or incorporation of artificial intelligence.

### Laboratory Tests

Laboratory testing interim guidance for SARS-CoV-2 has been brought to us by the World Health Organization (WHO) to diagnose the suspected cases (10). The recommended respiratory sample from the suspected patients should be collected, and the collection could be performed from the upper or lower respiratory tract. The oropharyngeal or nasopharyngeal swab could be collected for upper-respiratory samples from the ambulatory patients, whereas for patients with severe respiratory disease, sputum (if produced) and/or endotracheal aspirate or bronchoalveolar lavage need to collect for nucleic acid amplification tests. Blood samples should also be collected from the patients to define retrospectively. The paired samples from the same patient at the first and 2–4 weeks of exposure should be collected for confirmation. Collected samples should be stored at 2–8°C if the storage period is <5 days, and for longer storage, the samples should be kept at −70°C (dry ice).

Nucleic acid sequencing of RNA of the virus is a routine confirmation of the infection by nucleic acid amplification tests, which incorporate real-time reverse transcription–polymerase chain reaction (rt-PCR). The extracted samples of RNA are amplified by rt-PCR and assayed with specific primers and probes of SARS-CoV-2 using the WHO-designed protocols in different laboratories across the globe (27). All the tests are carried out in the recommended laboratories with all the facilities and safety measures in different countries. In the case of test-positive targets for RNA-dependent RNA polymerase (open reading frame 1ab) confirming the infection of SARS-CoV-2, a second confirmation could be achieved by the positive test in nucleocapsid protein (28, 29). Details of diagnostic protocols are available in the guidelines for the detection of the presence of pneumonic infection (10, 29). Repeated sampling and analysis are confirmatory of recovery from the infection. Alternatively, the analysis of paired serum from the collected blood samples of the infected patients also provides confirmation of infection (10).

### Imaging Investigations

Findings of computed tomography (CT) images vary in persons with different stages of infection at the time of infection, different ages, immunity status, presence of comorbidities, and drug interventions; however, CT imaging of the infected persons is strongly recommended for the diagnosis of the disease (26). Alternatively to CT images, ultrasound or radiograph of the lungs with bilateral opacities, presence of nodules, lobar or collapse, and volume overload may help in the diagnosis of the COVID-19, with its stages (30). The chest images would provide the characteristics of the lesions, which could be differentiated by the quantity, distribution, shape, density, and concomitant signs of the lesions. These images help to diagnose the patients at different stages of their infections, such as ultra-early stage (with no clinical manifestation of the patients with positive throat swab and negative laboratory test, when exposed to the virus-contaminated location within 1–2 weeks), early-stage (with clinical manifestations within 1–3 days), rapid progression stage (with the clinical manifestations for 3–7 days), consolidation stage (after 7–14 days of appearing clinical manifestations), and dissipation stage (2–3 weeks after the appearance of clinical manifestations). For a detailed understanding of the imaging characteristics, please refer to the cited article (26).

## Transmission Possibilities and Preventive Measures of COVID-19

**Figure 3:**
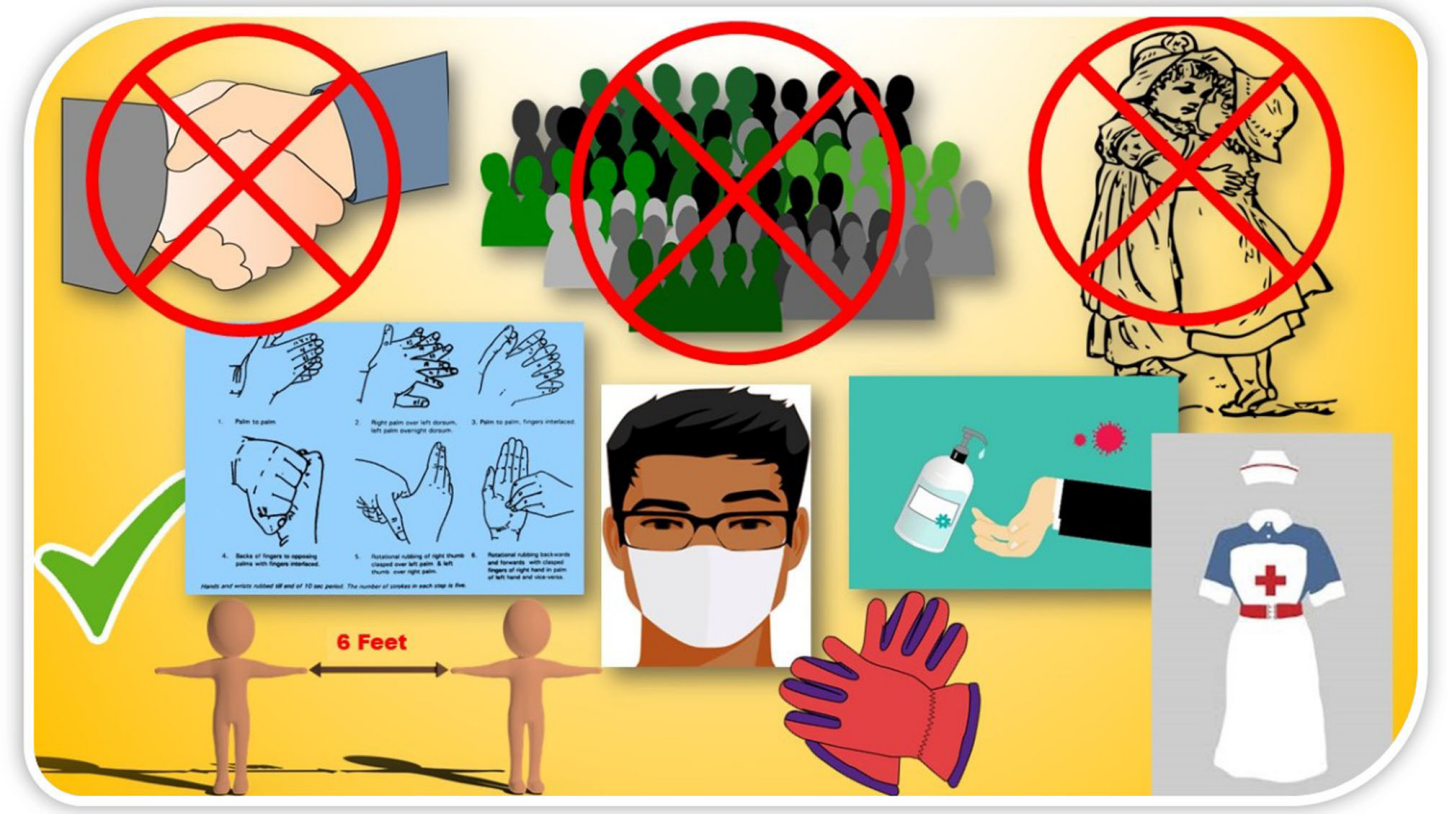
Do's and don'ts to protect yourself from COVID-19 infection.

During this critical stage of the pandemic outbreak of COVID-19, it is very much essential to control the spreading of infection by knowing and controlling all the possibilities of transmission patterns. Many reports throughout the world presented transmission dynamics in order to understand the transmission pathway of COVID-19. Even though the initial few cases of COVID-19 were of zoonotic (animal-to-human) origin, linked to the Wuhan seafood market, however, a thousand-folds increased cases point toward another source of transmission, including person-to-person (31). Recently, person-to-person transmission is one of the main concerns to control this pandemic situation, which is most commonly associated with close contact with COVID-19–infected person, where virus transmission can happen via respiratory droplets produced by the infected person upon coughing or sneezing to the mouth, eyes, or nose or directly to the lungs of the healthy person (32, 33). Sporadic imported cases, who visited China, not Wuhan wet market, have been reported in the last 4 months in different countries. In Vietnam, in one couple after visiting Wuhan, not Wuhan wet market, the 65-year-old husband with comorbid disease was reported to be CoV positive. Further, his healthy son within 3 days after spending time with him developed dry cough and fever and further after hospitalization tested positive, although his son had not visited Wuhan. Although it was not confirmed, however, his father was assumed to be the cause of infection (34). Person-to-person transmission is evident in a family cluster of six COVID-19–positive patients as well (35). The first reported case of COVID-19 in the United States, who visited Wuhan, however, had no time spent in Wuhan market as well as this person did not come in close contact with any ill patient; however, after 5 days of his return from Wuhan, nasopharyngeal and oropharyngeal swabs tested positive. Remarkably, COVID-19 virus RNA was detected in the stool sample, collected on day 7 of the patient's illness, whereas the serum sample tested negative (36). Even in China Focus News, it has been reported that top scientists isolated CoV from feces samples collected from infected patients. However, researchers urge further evidence to confirm fecal–oral transmission (37). Li et al., investigated and presented an estimate of *R*_0_ (number of cases directly produced from one susceptible case of COVID-19) until January 4, 2020, of 2.2, which represents that on average 2.2 persons have been infected from one COVID-19 patient. The group also alerted on the epidemic increase as an estimated *R*_0_ value was >1 and further suggested for control measure to reduce transmissibility (38). A similar representation of *R*_0_ value of 2.2 (90% high-density interval, 1.4–3.8) was reported by another group, and similarly, the team was suspected for a continuous person-to-person transmission with sustained transmission chains (39). Besides, a recent tweet by art director Gary Warshaw presented a graphic by Robert A. J. Signer (assistant professor, University of California San Diego), stating a single infected person can transmit to 2.5 people within 5 days; the number will increase to 406 infected people within the period of 30 days. Further power of social distancing in preventing COVID-19 transmission was explained; if there is 50% less exposure, infection possibilities can be reduced to 15 people within 30 days; further, it can be reduced to 2.5 people on further reduction of exposure to 75% (Figure 3).

Although most reported transmission through symptomatic carriers, however, asymptomatic carriers also become the potential source of COVID-19 (40–42). Possibilities of asymptomatic infection were presented in another report (35). Therefore, the effort to stop spreading COVID-19 will be challenging, if asymptomatic carrier transmission replicated (40).

Safety of healthcare providers from COVID-19 is one of the major challenges, because of irresistible COVID-19 cases throughout the world, which strains healthcare facilities and further extended adverse events to the healthcare providers. In China, around 3,000 healthcare providers have been infected, and among them, around 22 had died (43). Another report revealed hospital-acquired infection of 57 (41%) of 138 patients, where 40 (29%) were frontline healthcare providers (21). It has been reported that mortality from COVID-19 is more frequent in older people; however, Dr. Peter Hotez, National School of Tropical Medicine at Baylor College of Medicine, revealed that younger healthcare providers are even at greater risk of serious illness (44).

Uncontrollable spreading of COVID-19 could be prevented by generating public awareness, and thus, the Centers for Disease Control and Prevention (CDC) recommended safety measures for healthcare providers for the public. As mentioned earlier, SARS-CoV-2 is not an airborne virus; it is mainly transmitted by droplet to close contact; therefore, personal protective equipment as recommended by CDC, such as N95 facemask, gloves, gown, goggles/face shield, or powered, air-purifying respirator, can prevent transmission to the healthcare provider during proximity with the infected patient (32, 43).

Similar to other flu viruses, a SARS-CoV-2–infected patient can produce approximately 3,000 aerosol droplets following a single cough, which further can land on cloth or body surface of a healthy person, or surfaces. It has been reported that the virus in an aerosol droplet can survive within the experimental period of 3 h. Further, the same group reported that the virus can survive longer on plastic and stainless-steel surfaces (>72 h), copper (<4 h) and cupboard (<24 h) (45, 46). Thus, special precautions should be taken care to inactivate the virus on the surface by using household bleach containing 0.1% sodium hypochlorite, 62–71% alcohol, or 0.5% hydrogen peroxide. The concerned persons are using different useful techniques to avoid exposure such as frequent cleaning and sanitizing their hand, using masks when in public, opening doors using their elbows, avoiding grabbing handle vehicles, avoiding greeting by a handshake or hugging, and maintaining social distancing (46). We should continue these healthy practices and share with others using social media and/or journal article platform to fight together this pandemic outbreak.

## Treatment Progress Against COVID-19

The clinical signs and symptoms of the COVID-19 patients worsen during the incubation in an infected person. Initially, the condition would not be critical to hospitalize the patients immediately, according to WHO. However, during the progression of the infection to the lower respiratory tract, the condition may worsen during the second week of illness (47). All the suspected patients should be advised by the medical practitioners to be isolated in a single room or self-quarantined at home, whereas confirmed cases of COVID-19 must be admitted to the hospital wards. In case of the critical condition of the patient, it should be shifted to the intensive care unit immediately for proper care (48). Whatever the stage would be, all the patients should be monitored closely, where the severity of the progression may be influenced by older age and/or comorbid diseased conditions, such as immunocompromised conditions, lung disorder, heart failure, cancer, renal disease, cerebrovascular disease, liver disease, and pregnancy (47).

So far, there is no specific treatment available for the treatment of COVID-19; however, management of the disease demands prompt execution on prevention of the infection with control measures and supportive management of complications (47). As general treatment measures, the patients would be provided the facilities of complete bed rest with sufficient intake of calorie, water, and possible supportive treatments.

**Figure 4:**
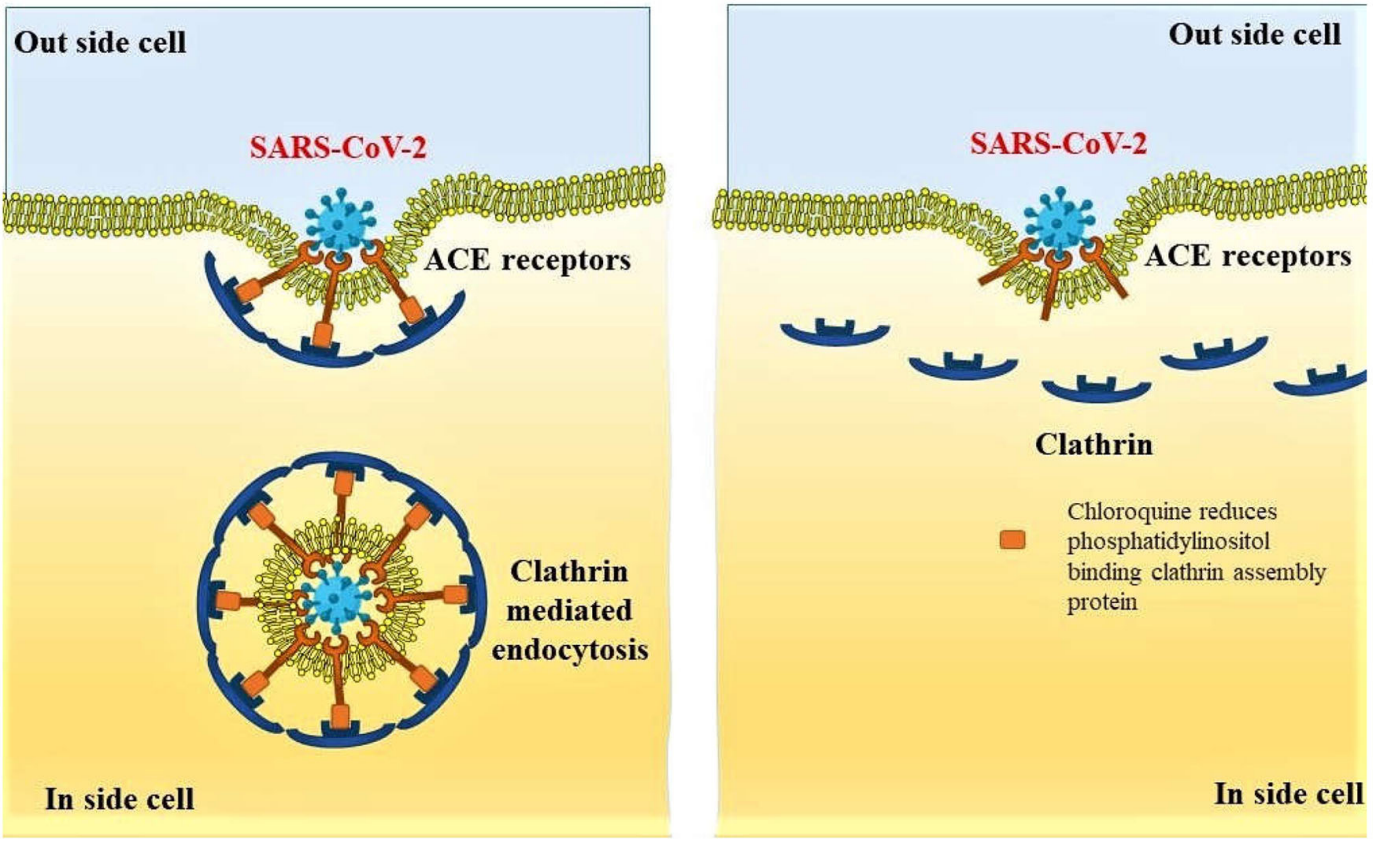
Preventive mechanism of SARS-CoV-2 endocytosis by decreased expression of phosphatidylinositol binding clathrin assembly protein, leading to reduced internalization of the viral particles.

Recent reports on oral treatment with antimalarial agents, chloroquine, and an antirheumatoid arthritis agent hydroxychloroquine have shown an early promise in the treatment of COVID-19. The *in vitro* efficacy of chloroquine was reported to stop the infection caused by SARS-CoV-2 at low concentration. The half-maximal effective concentration of this agent against the pathogenic agent is 1.13 μM; however, the 50% cytotoxic concentration of the drug is >100 μM (49). Antiviral efficacy of chloroquine has been explained by the increase in endosomal pH to prevent fusion between the virus and cell. Simultaneously, chloroquine impedes glycosylation of cellular receptors of the virus (50, 51). A recent report revealed another possible mechanism of chloroquine in the prevention of endocytosis of the SARS-CoV-2 viral particles where the authors mentioned that clathrin-mediated endocytosis of the viral particles is prevented by the decreased expression of phosphatidylinositol-binding clathrin assembly protein (Figure 4) within the cells (52).

The outcome of 21 clinical trials so far (to date) for the treatment of COVID-19–induced pneumonia had shown the safety and efficacy of chloroquine or hydroxychloroquine in more than 10 hospitals in different places of China (http://www.chictr.org.cn/searchprojen.aspx?title=hydroxychloroquine&officialname=&subjectid=&secondaryid=&applier=&studyleader=&ethicalcommitteesanction=&sponsor=&studyailment=&studyailmentcode=&studytype=0&studystage=0&studydesign=0&minstudyexecutetime=&maxstudyexecutetime=&recruitmentstatus=0&gender=0&agreetosign=&secsponsor=&regno=&regstatus=0&country=&province=&city=&institution=&institutionlevel=&measure=&intercode=&sourceofspends=&createyear=0&isuploadrf=&whetherpublic=&btngo=btn&verifycode=&page=1). Treatment with chloroquine phosphate had shown superiority in the treatment in the prevention of pneumonia in the COVID-19–infected patients (50). The clinical studies with these drugs are confined not only in China; around five of the studies already registered in the National Institutes of Health, US National Library of Medicine (https://clinicaltrials.gov/ct2/show/NCT04286503?term=Chloroquine&cond=COVID&draw=2&rank=4). Treatment of COVID-19 infection with chloroquine phosphate did not exert a serious adverse effect on the users, and there are no shreds of evidence of cardiotoxicity, but reported to promote negative conversion and reduction in disease progression, as evidenced by the radiographic images of the lungs (50, 53). Although not approved, the progress of research had suggested the use of these drugs for the control of this pandemic outbreak (53).

A recent short clinical report on the combination of hydroxychloroquine and azithromycin on 36 COVID-19 patients revealed a sharp reduction of viral load in the upper-respiratory samples from infected patients within 6 days. The clinical benefits of this combination are yet to be reported; however, patients with chronic disease such as renal failure or hepatic disease or those receiving medications for arrhythmia need to take precaution with prescribing this combination, as there might be possibilities of QT prolongation. The authors suggested that the efficacy of hydroxychloroquine in the reduction of viral load could be significantly improved when combined with azithromycin (53, 54).

A combination of antiviral agents had shown potential in the management of the COVID-19 outbreak. Several nucleoside analogs (investigational: galidesivir and remdesivir and approved: ribavirin and favipiravir) are in the different stages in the clinical research. These nucleoside analogs prevent the RNA-dependent RNA polymerase and thereby inhibit viral RNA synthesis in the virus particles (17). Among the investigational drugs, remdesivir gained attention in the management of the critical pneumonic condition of COVID-19 patients. This broad-spectrum antiviral agent is intended to be administered intravenously, which terminate RNA transcription prematurely of the SARS-CoV-2, thereby inhibiting the replication of the viral genome. It has well-established activities against beta-CoVs and *in vitro* control against novel COVID pathogen (EC_50_ = 0.77 μM in Vero E6 cells) (49, 55, 56). There are several clinical researches recruiting COVID-19 patients in different stages of the clinical trial for the establishment of this investigational agent for the treatment of SARS-CoV-2 (https://clinicaltrials.gov/ct2/results?term=Remdesivir&cond=COVID).

Efficacy of the guanine-analog influenza agent, favipiravir (T-705), had shown its potential against several viruses, including SARS-CoV-2 (EC_50_ = 61.88 μM in Vero E6 cells) (49). Considering the efficacy of this nucleoside analog against COVID-19, a combination of interferon-α and favipiravir is used to evaluate the efficacy and safety in a randomized trial. The recruitment of the trial is ongoing, where the other groups are receiving interferon-α and combinations of interferon-α, lopinavir and ritonavir (human immunodeficiency virus protease inhibitor antiretroviral agents) (ChiCTR2000029548) (57). Clinical trial on the combination of an approved influenza inhibitor, baloxavir marboxil, and favipiravir is also ongoing against COVID-19 (ChiCTR2000029544). Further, this favipiravir is combined with chloroquine phosphate (ChiCTR2000030987) and toclizumab, an immunosuppressant (ChiCTR2000030894) is also registered to be evaluated for the treatment of this pandemic pneumonia. Alternatively, the clinical trials on the combination of lopinavir and ritonavir are also continuing against mild cases of COVID-19 infection (ChiCTR2000029539). Additionally, the effectiveness of ribavirin is also under investigation in combination with lopinavir/ritonavir plus interferon-α (ChiCTR2000029387) (58).

Alternatively, SARS-CoV-2 enters into the lung cells by the ACE2 receptor–mediated entry, particularly through the AT2 cells of the lungs (59). This endocytosis process is influenced by the AP2-associated protein kinase 1 (AAK1). Among the 47 ligands approved for the control of different diseased conditions, 6six of them are known to have a higher affinity toward AAK1. Considering the safety of the compounds, baricitinib is suggested to be an important agent for the control of SARS-CoV-2 endocytosis within the host cells (60); however, clinical evidences are still necessary to support this treatment (61).

There is no vaccine available for SARS-CoV-2 infection yet; however, continual research has brought an investigational vaccine (mRNA-1273) for protection against COVID-19 in a phase I clinical trial. This research is initiated at Kaiser Permanente Washington Health Research Institute at Seattle, which is developed by the National Institute of Allergy and Infectious Diseases and its collaborator Moderna, Inc. The enrolment of subjects is ongoing for 45 healthy volunteers, where the first volunteer received the vaccine on March 16, 2020 (NCT04283461) (62). Another vaccine has been developed by Shenzhen Geno-Immune Medical Institute and under investigation under phase I clinical trial, where the vaccine is an engineered minigene with an efficient lentiviral vector system. The vaccine will express viral proteins and immune-modulatory genes to transform artificial antigen-presenting cells and to activate T cells (NCT04299724). A similar trial by Shenzhen Geno-Immune Medical Institute is ongoing with a synthetic vaccine to target and modify immune-modulatory dendritic cells with activation of T cells (NCT04276896). Another clinical research has been registered by CanSino Biologics Inc., for recruitment of healthy volunteers to assess safety, reactogenicity, and immunogenicity of the recombinant novel CoV vaccine (adenovirus type-5 vector) (NCT04313127). It could be said that the persistent research on small molecules and vaccines may bring novel compounds for the treatment or prevention of the disease in an effective manner to eradicate this pandemic threat.

## Progress of Vaccine Development Against COVID-19

We are at the ninth month of disease outbreak and already covered 6 months from its announcement of pandemic. The researchers explored widely on structural biology and genomics of the viral particle during this time in order to bring a new sunrise against this COVID-19. Development of vaccine is a tiresome, lengthy, and costly process where the rate of attrition is high (63). Development of a licensed vaccine usually takes multiple candidates and several years; still there is not much evidence on the cost on development (64). However, the developmental process during the pandemic paradigm does not follow linear sequential steps of development. There will not be multiple pauses for investigating process check and analysis of data. Instead, a faster path is accomplished through parallel execution of different steps together; for example, preclinical studies can be run in parallel with the phase I clinical study (63).

Several challenges had been reported against development of vaccine against SARS-CoV-2. It has been observed that the spike protein on viral surface is immunogenic; however, designing suitable antigen is a critical task to obtain immune response against the pathogen. Previous experience toward development of SARS and MERS vaccine has raised alarms toward aggravating respiratory problems in preclinical studies. Such conditions might be a result of antibody-dependent action or direct action on the lungs by the vaccine candidates. Thus, it is necessary to perform rigorous monitoring of safety profile in suitable animal model. Alternatively, the duration of immunity development in people is also needed to ascertain for proper protection; otherwise, the same pathogen may affect the immunized person after certain period (65, 66).

However, continuous effort by researchers has brought more than 170 vaccine candidates into research by various pharmaceutical institutions and industries (67). Numbers of vaccines among those have crossed the barriers of laboratory and entered into different stages of clinical trials. According to the latest report (August 31, 2020), 49 vaccines are at phase I to early/limited use in human subjects for immunization. More elaborately, 23 candidates are in phase I, 14 candidates are in phase II, nine candidates are in phase III, and three of them have received approval for early/limited use in human subjects for immunization (68). One of the three is developed by CanSino Biologics, China. This product has entered into phase III on August 2020, and trial has been started in Saudi Arabia and Pakistan. Gamaleya Research Institute, Russia, developed the other vaccine, which was named Sputnik V by the Russian healthcare regulator. Sinovac Biotech, China, developed the third approved vaccine for early/limited use, CoronaVac. This vaccine had received an emergency approval by the Chinese Government for limited use in July 2020. However, none of the vaccine has received full approval so far for immunization of human against COVID-19.

Moderna Therapeutics is the first biotechnology company that has brought a vaccine against COVID-19, where the vaccine contains messenger RNA, which will produce viral proteins in the body. Their studies are progressing and currently at phase III of clinical research (67). Exceptionally speedy research to fight against COVID-19 has become possible by quick recognition of the viral particle and identification and determination of genetic sequencing (69). Overcoming the barriers of scale-up process of protein-based products, several vaccines are in production stages to be tested in large numbers of people to protect the world against this deadliest SARS-CoV-2.

## Global Economic Impact of the COVID-19

After the most serious global health crisis of Spanish flu in 1918, COVID-19 is the most economically costly pandemic in the world. After the declaration of COVID-19 as a world health emergency by WHO, it has affected almost $90 trillion of global economy (70). Initially, there was no much coordination between many countries to combat COVID-19 pandemic. After the G-7 emergency meeting, countries pledged to combat the pandemic by coordinating research efforts, enhancing medical equipment availability, monetary and fiscal measures, mobilization of policy instruments, and lastly targeted actions to support sectors, companies, and support workers affected by the spread of COVID-19. Later, the G-20 has a broader membership, viewed in the response to the global financial crisis, and gained momentum on debt relief for low-income countries. The G-20 includes the G-7 countries plus Argentina, Australia, Brazil, China, India, Indonesia, Mexico, Russia, Saudi Arabia, South Africa, South Korea, Turkey, and the European Union (71).

COVID-19 caused sudden economic disruption in almost every area of human endeavor. The most affected are travel industry, hospitality industry, sports industry, event industry, entertainment industry, education sector, oil-dependent countries, import dependent countries, financial sectors (banks and Fintech), financial markets, etc. (72). Some important aspects in different industries are discussed in the connecting section of the article.

### Travel Industry Sector

Since the inception of CoV pandemic, tourism is the first industry that has fallen immediately. The small viral particle has halted millions of trips around the globe. The imposition of travel restrictions by many countries affected indefinite suspension of tourism travel, visas on tourism, work, business, etc. Many countries imposed travel bans (both inward and outward travel) and shut down their international airports. Mass passenger cancelation led to business loss because of empty flights. Most countries' famous airline industries are already shut, and many employees lost their jobs. So far, it has been estimated that there may be more than $820 billion revenue loss in the business travel sector alone because of the COVID-19 pandemic (73, 74). However, the presented data are changing every moment as the virus is spreading. It has been estimated during the announcement of COVID-19 as a pandemic that there might be 75 million loss of employment with $2.1 trillion loss in revenue from the tourism sector (75).

### Hospitality Industry Sector

To combat the pandemic, many governments announced important messages such as “social distancing, stay home” policies and movement restrictions. These restrictions led to shutdown of many businesses of street hawkers, restaurants and small and star hotels, pubs, and other related entertainment outlets. Many hotels witnessed no customers and also faced booking cancelations. Staffs who are working in these premises lost their jobs, and many owners of these outlets announced suspension of normal operations and became bankrupts (76). Governments are also providing supportive measures to the hospitality industry, such as “Eat Out to Help Out” program in the United Kingdom for 1 month, where 100 million meals were dispensed by the 84,700 restaurants (77). A very few hotels are converted to quarantine COVID patients, but the proportion is negligible (76). Additionally, there are offers to pay 50% of the diner's bill for the food and soft drinks, which helps to retain their staffs employed and helps to survive the hospitality farms (77).

### Sports Industry Sector

Sports industry is one of the severely affected industries. Almost all the sports are either suspended or postponed in many parts of the world. Because of COVID outbreak, many important football leagues such as England, Scotland, European, and Turkish leagues were canceled or suspended. Olympic and Paralympic games scheduled in Tokyo got postponed. Formula One and Grand Prix were canceled, along with England's FIH Pro League games. There are plenty of other matches related to rugby, baseball, motorsports, snooker, swimming, golf, and other indoor and outdoor games either suspended or canceled. The organizers and sponsors lost their revenue in billions of dollars. In addition, many players also lost their income (72).

### Impact on the Event Sector

The event sector is an important contributor to the economy. Business events generated more than $1.07 trillion of direct spending. These business events involved more than 1.5 billion participants across more than 180 countries. In addition, these events supported more than 10.3 million direct jobs globally (78). Many important events such as business exhibitions, conferences, weddings, live music shows, corporate events, parties, product launches, trade shows, etc., are largely hit. Some of the events are canceled or rescheduled or conducted online. Few famous ticketing agents or companies such as Eventbrite canceled many events and severely affected their business.

### Entertainment Industry Sector

The International Alliance of theatrical stage employees reported that the global film industry incurred a huge loss in this outbreak. Millions of people lost their jobs. COVID outbreak severely affected various sectors such as the film screening sector, theater segment, live music segment, dance and stage performance segment, drama segment, etc. According to Broadcasting, Entertainment, Communications and Theater Union, unemployment levels rose to unprecedented high. Many Hollywood and regional movie productions were postponed or canceled indefinitely, and even the theaters and cinema owners are also affected because of poor audiences (79).

### Pharmaceutical Industry Sector

There is a huge impact on the manufacturing of pharmaceutical and related industries too. The outbreak affected the pharmaceutical supply chain. Because of this, pharmaceutical companies around the world relied heavily on China for both active pharmaceutical ingredients (APIs) and excipients. Around 60% of pharmaceutical excipients and APIs were manufactured and supplied by China. Because of the CoV outbreak, all the manufacturing industries of excipients and APIs were closed down, and both manufacturing and supply chain were severely affected. Many manufacturing companies did not store enough of excipients and APIs before the CoV outbreak, and it severely impacted those companies and ultimately impacted the whole healthcare sector (80).

### Education Sector

The majority of the governments around the world instructed schools, colleges, and universities to shut down because of this pandemic. Educators, teachers, students, parents, and all other related stakeholders were severely affected morally, financially, and psychologically and all other sorts. The pandemic effect had severe consequences on schools, colleges, and universities where there are no online learning platforms. Teachers, students, and administrators have a difficult time adapting to and adopting the technical, financial, and academic changes required to cope with the new teaching and learning strategies. More than 300 million students were affected, and in many poor countries, online education for those students was a nightmare. The radical shift from traditional classroom education to online platforms was a huge challenge for both teachers and students. Low-income countries were suffering from the affordability, technology, change management, and adaptability to online teaching (81).

Thus, COVID-19 trims global economic growth by 3.0–6% in 2020 with a partial recovery in 2021. This COVID-19 global recession is distinctive and differs from previous episodes in that global synchronized lockdowns and disturbance of financial markets strengthen one another into record economic sudden stop (71).

Table 2 highlights how the various industries are affected and approximate loss due to COVID-19. COVID-19 has created a revolution on both domestic and multinational companies to strategically approach their business models. This challenging situation has forced companies to adapt to present challenges, day-to-day operations, managing workforce, adhering to government mandates, and finally reacting to customer and employee needs. Many firms grab the opportunity of digitization. Firms are now agile and developing their capabilities to survive like education institutes. Yes, there is no doubt some companies are closed down, some are still running under loss, and some are striving hard in transition. Once the crisis subsides, “true economic value once again becomes the final arbiter of business success” (88).

**Table 2:** Global economic impact on different sectors due to the outbreak of COVID-19.

**Industry**	**What is affected?**	**Approximate loss**
Travel industry	•Essential travel ban •Tourism travel ban •Ban of work and immigrant visas •Shutdown of airports •Flew of empty flights •Suspensions of airlines	Aviation industry loss—$113 billon (73) GTBA business travel sector—$820 billion (82)
Hospitality industry	•Restaurant business •Shutdown of cities and states •Cancelation of hotel bookings •Staff laid off and shutdown of business	Booking cancelations $150 billion (83)
Sports industry	•Suspension of football leagues, Formula one •Olympic and Paralympic games •Hockey, cricket, rugby, baseball, motors sports, snooker, swimming, golf, etc. •Loss of revenue to the sponsors and organizers	Huge amount of loss which is difficult to estimate and recover (84)
Event industry	•Cancelation of events (live shows, exhibitions, conferences, parties, corporate events, trade shows, etc. •Business event-related travel, event ticketing segment •Direct spending by exhibitors •Millions of direct job loss	More than 1.07 trillion dollars (78)
Entertainment industry	•Postponement of Hollywood movies •Goodbye to cinema and theaters •Job loss of millions •Cancelation of movie shoots, shows	More than $5 billion (85)
Education sector/industry	•Closure of schools, universities •Disruption of 290.5 million students (86) •Unemployment in the sector •Cancelation of overseas travel and abroad programs of foreign students	More than $600 billion (87)
Financial market	•Effect on global stock market •Fall in stock market indices •Effect on share price	Global stock market lost $6 trillion from February 23 to 28 alone (72)

## International Cooperation Toward Combating COVID-19 Lead Economic Impact

The most significant threats the world is facing currently are climate change, terrorism, cybercrime, and infectious diseases. This COVID pandemic is a powerful reminder of interconnectedness and vulnerabilities, and it has made one thing clear—that we are one human family, and the only way to mitigate is to work collaboratively. There are various platforms such as G7, G20, WHO, IMF World Bank, and many other international organizations to address the acute health, economic, and social consequences of the pandemic crisis. Recently, the European Union hosted an online summit where around 40 countries took part and pledged to help develop a CoV vaccine. A large-scale and science-based global response that is transparent and robust is required in the spirit of solidarity. Cooperation among countries is not only ethical and imperative but also an existential one.

The pandemic outbreak is causing havoc in rich countries, and the damage caused in poor countries is often overlooked. The most worrying part is that the economic crisis is not confined to one region or country; it is happening across the world. The global economy shrunk faster than at any time since World War II. This is a very crucial time, and all the developed and developing nations should come forward and support each other in whatever way possible. There are plenty of issues to address, such as raising infection rates, weak healthcare systems, massive economic damage, unemployment, disruption in supply chain, poverty, famine, etc. Hence, there is a need for an urgent global plan to address the important issues such as the following:

- There is a need for external funding to address massive economic stimulus packages to revive the economy and reduce unemployment, prevent starvation, and address poverty.
- The global plan should assist in stabilizing their currencies.
- An immediate moratorium should be in place for poor countries whose debts are mainly to western governments. It is of absolute importance to assist the most vulnerable countries such as Africa. In addition, foreign-held private debt collection needs to be halted temporarily for at least 3–5 years.
- To limit the spread of pandemic, immediate assistance is needed to strengthen the healthcare systems by increasing needs of medical supplies; exchange epidemiological and clinical data; sharing necessary materials for research and development; implementation of WHO health regulations; discovery and making use of vaccine at an affordable price on an equitable basis to the world population; and preventing the spread of other infectious diseases for a period at least 3–5 years (89).

To address the above, it may cost around $2.5 trillion. At the global level, institutions must be given the authority and resources to deal with this pandemic. Global institutions that could organize the response but need more resources such as World Bank, IMF, and WHO are being hobbled by western governments (90). Populism and xenophobia have gained ground nowadays; these can be addressed only by boosting the legitimacy and effectiveness of global institutions.

## Conclusion and Future Remark

This pandemic outbreak of COVID-19 has brought a tough time for the healthcare providers where the confirmed SARS-CoV-2 cases are diagnosed with mild to severe respiratory conditions to death. Thus, the rapid identification of the invasive fatal particles is the major challenge for this fast-spreading outbreak. Transmission could be prevented by the preventive measures specified by WHO, where several existing and investigational compounds are at different stages of clinical research. Several healthcare professionals are working effortlessly throughout the world to find promising ways toward effective control against this pandemic situation. Few of the tested therapeutics, such as hydroxychloroquine, remdesivir, and some other antiviral agents, has shown positive response against the treatment of the disease; however, treatments are prescribed under medical supervision for safety concern.

Various measures had been taken to prevent the transmission of the virus to the healthy person, such as complete/partial lockdown of the countries, maintaining social distancing and using protectives, isolation of the identified patients, quarantine of the suspect, extensive screening and identification of COVID-19–positive cases, contact tracing to find the cross-infected personnels; etc. Despite the several measures being taken by different countries, the scale of rising daily new cases is still sharp; hence, people are required to be more concerned toward control of this condition. Separate trained teams need to be formed to handle the infected cases effectively, whereas government aids toward novel research against combating this situation are highly needed. With the unremitting effort by researchers and healthcare providers, we pray for a SARS-CoV–free world to breathe freely with a new sunrise.

## Author Contributions

BG: writing—original draft. PK: writing—review & editing. RA: resources. NM: visualization. HC: supervision. All authors contributed to the article and approved the submitted version.

## Conflict of Interest

The authors declare that the research was conducted in the absence of any commercial or financial relationships that could be construed as a potential conflict of interest.
